# Development of a characterised tool kit for the interrogation of NLRP3 inflammasome-dependent responses

**DOI:** 10.1038/s41598-018-24029-3

**Published:** 2018-04-04

**Authors:** Elena Redondo-Castro, Dorte Faust, Simon Fox, Alex G. Baldwin, Simon Osborne, Michael J. Haley, Eric Karran, Hugh Nuthall, Peter J. Atkinson, Lee A. Dawson, Carol Routledge, Stuart M. Allan, Sally Freeman, Janet Brownlees, David Brough

**Affiliations:** 1 Division of Neuroscience and Experimental Psychology, School of Biological Sciences, Faculty of Biology, Medicine and Health, Manchester Academic Health Science Centre, University of Manchester, AV Hill Building, Oxford Road, Manchester, M13 9PT UK; 2 LifeArc, Accelerator Building, SBC Campus, Stevenage, SG1 2FX UK; 3 Division of Pharmacy and Optometry, School of Health Sciences, Faculty of Biology, Medicine and Health, Manchester Academic Health Science Centre, University of Manchester, Stopford Building, Oxford Road, Manchester, M13 9PT UK; 4 Abbvie, Foundational Neuroscience Centre, Cambridge, Massachusetts USA; 5 Eli Lilly Research Centre, Windlesham, Surrey GU20 6PH UK; 6 Eisai Limited, European Knowledge Centre, Hatfield, Herts AL10 9SN UK; 7 Astex Pharmaceuticals, 436 Cambridge Science Park, Cambridge, CB4 0QA UK; 8 Alzheimer’s Research UK, 3 Riverside, Granta Park, Cambridge CB21 6AD UK

## Abstract

Inflammation is an established contributor to disease and the NLRP3 inflammasome is emerging as a potential therapeutic target. A number of small molecule inhibitors of the NLRP3 pathway have been described. Here we analysed the most promising of these inhibitor classes side by side to assess relative potency and selectivity for their respective putative targets. Assessed using ASC inflammasome-speck formation, and release of IL-1β, in both human monocyte/macrophage THP1 cells and in primary mouse microglia, we compared the relative potency and selectivity of P2X7 inhibitors, inflammasome inhibitors (diarylsulfonylurea vs. the NBC series), and caspase-1 inhibitors. In doing so we are now able to provide a well characterised small molecule tool kit for interrogating and validating inflammasome-dependent responses with a range of nanomolar potency inhibitors against established points in the inflammasome pathway.

## Introduction

**Figure 1:**
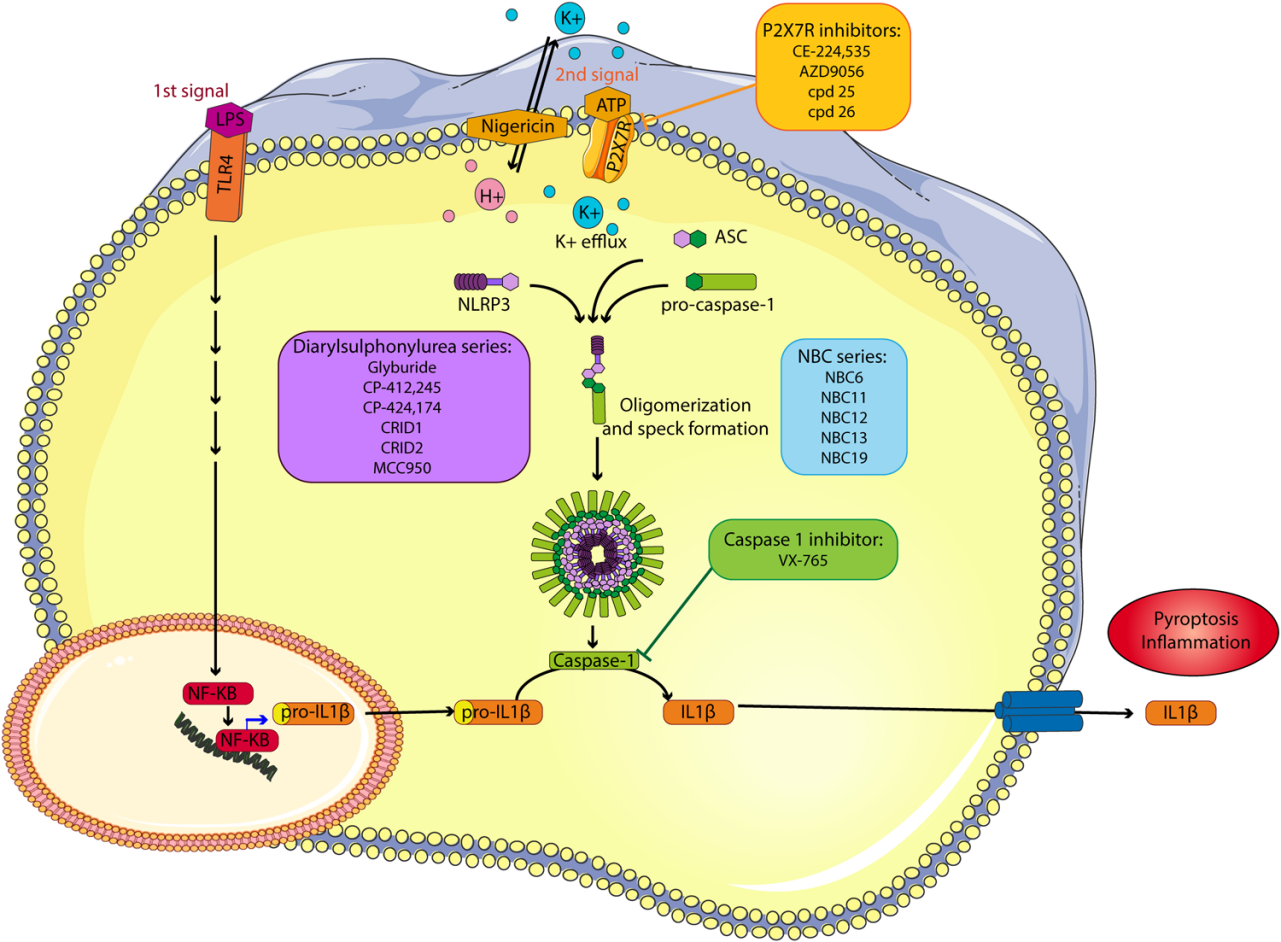
Inflammasome pathway and inhibitors. The action of LPS on TLR4 induces the translocation of NFκB to the nucleus and triggers the transcription of pro-IL-1β and NLRP3. A second signal (e.g. ATP acting at P2X7), causes NLRP3, ASC and pro-caspase-1 to oligomerize and form an inflammasome speck, which permits the recruitment and activation of caspase-1 and the subsequent cleavage of pro-IL-1β into its active form which is then released. The inhibitors were added directly before the second signal, and were characterised as P2X7 receptor inhibitors, a caspase-1 inhibitor, or the NLRP3 inhibiting diarylsulfonylurea and NBC series inhibitors. The outline of the cell is courtesy of Servier Medical Art.

Inflammation is a protective host response to infection, but when it occurs during non-communicable disease it is often damaging and contributes to an acceleration of pathology and a worse outcome. An important inflammatory process in disease is the activation of a multi-molecular complex called the NLRP3 inflammasome (Fig. 1)^1^. The NLRP3 inflammasome consists of a pattern recognition receptor (PRR), which in this case is NLRP3 (NOD-like receptor (NLR) family, pyrin domain–containing protein 3 (NLRP3)), an adaptor protein called ASC (apoptosis-associated speck-like protein containing a caspase activation and recruitment domain (CARD)), and pro-caspase-1^2^. Described mainly in cells of haematopoietic lineage NLRP3 requires priming by pathogen associated molecular patterns (PAMPs) and subsequently becomes activated by a further PAMP, or by damage associated molecular patterns (DAMPs) causing a disruption to cellular homeostasis^1^. A commonly described DAMP activating NLRP3 is high levels of extracellular ATP which is sensed by the cell surface P2X7 receptor^3^. Activation of P2X7 induces efflux of K^+^ causing the association of the protein NEK7 (never in mitosis A-related kinase 7) to NLRP3 facilitating its activation^4^. Active NLRP3 then nucleates the oligomerisation of ASC molecules into inflammasome ‘specks’ which provide the platform for the proximity-induced auto-activation of caspase-1^5^. Caspase-1 then cleaves the cytokine precursor molecules pro-IL-1β and pro-IL-18 to active molecules which are then secreted through an unconventional secretory route involving gasdermin D pores to the extracellular space where they drive inflammation^6–8^. Once formed the ASC specks can also be released and are stable in the extracellular environment where they further propagate inflammatory processes^9,10^.

The NLRP3 inflammasome and IL-1β are implicated in diverse and major diseases including Alzheimer’s disease^11,12^, diabetes^13^, cardiovascular disease^14^, and many others. The importance of IL-1β to disease was highlighted following the recent publication of the CANTOS trial, where patients with a history of myocardial infarction were treated with canakinumab, a monoclonal antibody targeting IL-1β^15^. In the CANTOS trial it was found that canakinumab treatment reduced the rate of recurrent cardiovascular events, and cancer mortality, in addition to many other clinical outcomes^15^. However, biologicals such as canakinumab may not be suitable for the treatment of diseases where penetrance across the blood brain barrier is important, and so a small molecule inhibitor of NLRP3/IL-1 is desirable.

A number of small molecule inhibitors for the P2X7-NLRP3-Caspase-1 axis have been described^16^. The aim of this research was to take a selection of what we considered to be the most promising lead compounds from the literature. We focussed our study on known and potent molecules for defined points in the pathway which included antagonists of the P2X7 receptor (CE-224,535^17^, AZD9056^18^, and two 5,6-dihydro-[1,2,4]triazolo[4,3-a]-pyrazine P2X7 antagonists (compounds 25 and 26 from^19^), the diarylsulfonylurea series (glyburide through to the cytokine release inhibiting drugs (CRIDs), including MCC950^20–22^), the caspase-1 inhibitor belnacasan (VX-765)^23^, and compare these to several analogues of the recently described Novel Boron Compound (NBC) inflammasome inhibitor series of boron-containing inhibitors^24^ (Fig. 1). This selection of molecules is by no means comprehensive and it is important to acknowledge the recent development of additional NLRP3 inhibitors not tested here such as CY-09^25^, and OLT1177^26^. All molecules were tested in pre- and post-differentiated human macrophage THP1 cells using ASC speck formation and IL-1β release as endpoints, and in primary cultures of mouse microglia using IL-1β release as an endpoint. Thus we are now able to provide quantitative and comparable data for some of the most potent P2X7-NLRP3-caspase-1 inhibitors available.

## Results

**Figure 2:**
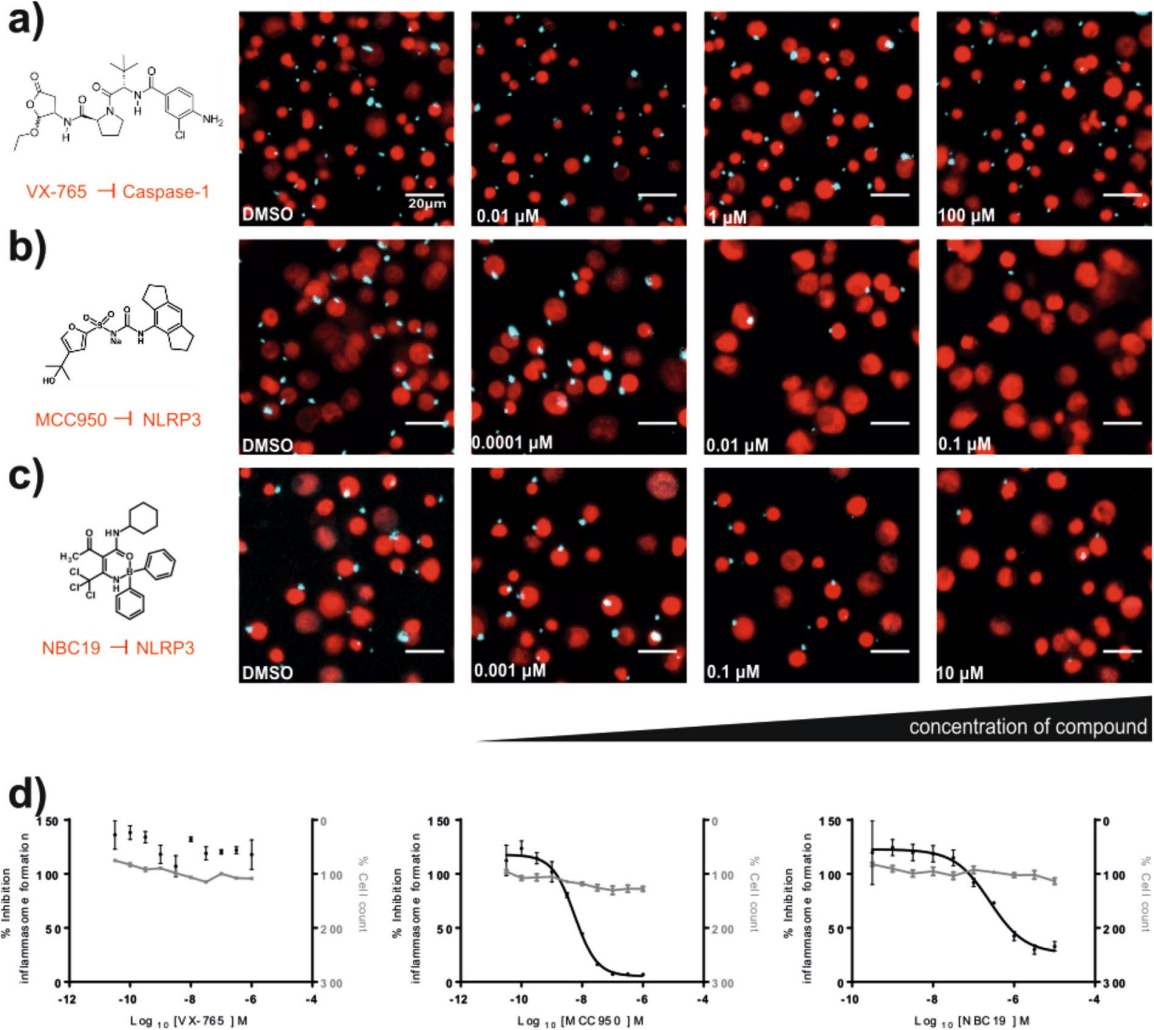
Compounds targeting NLRP3 but not downstream caspase-1 inhibit nigericin-induced formation of ASC specks in THP1-ASC-Cerulean macrophages. THP1-ASC-Cerulean macrophages were seeded in 96 well plate format, differentiated with PMA (0.5 µM, 3 h), pre-treated with compound for 30 min (VX-765 (**a**), MCC950 (**b**) and NBC19 (**c**)) before being stimulated with nigericin (10 µM, 1 h), fixed and imaged on GE InCell2000. Specks can this way be visualized and counted. Compounds targeting the NLRP3 inflammasome assembly induced a dose-dependent reduction in the number of specks. Dose response curves (**d**) showing the number of formed specks and the cell death for each compound. Scale bar depicts 20 µm. Respective concentration of compound and DMSO controls are indicated in the images. Data represent 3 separate cultures.

The structures of the molecules tested in the assays reported below are shown in Table 1. Human THP1 cells stably expressing ASC-Cerulean^9^ were either left undifferentiated or differentiated with phorbol 12-myristate 13-acetate (PMA, 0.5 µM, 3 h) before priming with bacterial endotoxin (LPS, 1 µg/ml overnight). Cells were then treated with the pan-caspase inhibitor ZVAD (50 µM, 30 min) to prevent pyroptosis and 10 point concentration response curves for each inhibitor were generated in triplicate measuring ASC speck formation in response to the K^+^ ionophore nigericin (10 µM, 1 h) using the IN Cell Analyzer 2000 imaging system (Fig. 2, and see methods). Under the current experimental conditions the THP1 cells failed to respond to ATP (up to 10 mM), so P2X7 inhibition was not evaluated in these cells. The results of the screen can be seen in Table 2. From the diarylsulfonylurea inflammasome inhibitor series (including glyburide, CP-412,245, CP-424,174^20^, CRID1, CRID2^21^, and MCC950^22^), the most potent inhibitor of ASC speck formation was MCC950 with an IC_50_ of 3 nM in undifferentiated THP1 cells (Table 2). From the NBC inflammasome inhibitor series^24^ the best inhibitor was NBC19 with an IC_50_ of 60 nM based on speck formation in differentiated THP1 cells (Table 2). The caspase-1 inhibitor VX-765 had no effect, as expected, as the formation of the ASC speck does not require caspase-1 (Table 2). The above experiment was repeated in differentiated THP1 cells except without ZVAD and with IL-1β release as the endpoint (Table 3). Under these conditions the best inhibitor from the diarylsulfonylureas was still MCC950 with an IC_50_ of 4 nM (Table 3). NBC19 was still the best of the NBC series with an IC_50_ of 80 nM for inhibiting nigericin induced IL-1β release, and in this assay VX-765 inhibited IL-1β release with an IC_50_ of 10 nM (Table 3).

**Table 1:** Inhibitor series characterised and compared in this study.

Drug	Structure	Reference
*P2X7 antagonists*		
CE-224,535		^17^
AZD9056		^18^
25 (from [1]		^19^
26 (from [1]		^19^
*Diarylsulfonylurea inflammasome inhibitor series*		
Glyburide		^20^
CP-412,245		^20^
CP-424,174		^20^
CRID1		^21^
CRID2		^21^
MCC950		^21,22^
*NBC inflammasome inhibitor series*		
BC7		^38^
BC23		^39^
NBC6		^24^
NBC11		^24^
NBC12		^24^
NBC13		^24^
NBC19		^24^
*Caspase-1 inhibitor*		
VX-765		^23^

Drugs targeting different points of the NLRP3 pathway assessed in this study. Shown is the name, structure, and original reference for the compounds tested. Compounds tested belong to one of 4 inhibitor classes: P2X7 receptor inhibitors, diarylsulphonylurea inhibitors, NBC series inhibitors, and a caspase-1 inhibitor.

**Table 2:** Inhibition of nigericin-induced ASC speck formation in pre- and post-differentiated THP1 cells.

Drug	ASC speck formation in pre-differentiated THP1 cells		ASC speck formation in post-differentiated THP1 cells	
*Diarylsulfonylurea inflammasome inhibitor series*	*pIC*_50_	*IC*_50_ *(μM)*	*pIC*_50_	*IC*_50_ *(μM)*
Glyburide	5.4	4.22	6.1	0.89
CP-412,245	7.6	0.03	6.9	0.12
CP-424,174	7.7	0.02	7.5	0.03
CRID1	7.6	0.02	7.6	0.02
CRID2	8.1	0.01	8.4	0.004
MCC950	8.6	0.003	8.3	0.005
*NBC inflammasome inhibitor series*				
BC7	5.5	2.82	5.5	3.04
BC23	5.2	6.35	4.8	14.4
NBC6	5.2	6.31	5.3	5.06
NBC11	6.1	0.83	5.8	1.69
NBC12	5.9	1.3	6.2	0.6
NBC13	5.9	1.13	6.8	0.15
NBC19	5.6	2.41	7.2	0.06
*Caspase-1 inhibitor*				
VX-765	—	—	—	—

Cells were primed with 1 µg/ml LPS overnight, treated with inhibitors for 30 minutes and further stimulated with nigericin (10 µM, 1 h). In pre-differentiated cells caspases were inhibited by Z-VAD-FMK (Supernatants of post-differentiated cells were harvested for MSD quantification of IL-1β (Table 3)). pIC_50_ and IC_50_ values were determined by vehicle-normalised quantification of cells showing ASC specks, and obtained from at least 3 independent experiments.

**Table 3:** Inhibition of nigericin and ATP-induced IL-1β release from post-differentiated THP1 cells and primary cultured neonatal microglia.

Drug	Nigericin-induced IL-1β release from post-differentiated THP1 cells		Nigericin-induced IL-1β release from neonatal microglia		ATP-induced IL-1β release from neonatal microglia	
*P2X7 antagonists*	*pIC*_50_	*IC*_50_ *(µM)*	*pIC*_50_	*IC*_50_ *(μM)*	*pIC*_50_	*IC*_50_ *(μM)*
CE-224,535	—	—	—	—	6.18	0.66
AZD9056	—	—	—	—	7.52	0.03
25 (from^19^)	—	—	—	—	7	0.1
26 (from^19^)	—	—	—	—	7.52	0.03
*Diarylsulfonylurea inflammasome inhibitor series*						
Glyburide	6	1.12	4.68	20.81	4.57	26.86
CP-412,245	7.3	0.06	6.51	0.31	6.85	0.14
CP-424,174	7.9	0.01	6.51	0.31	6.92	0.12
CRID1	7.6	0.03	6.72	0.19	6.8	0.16
CRID2	8	0.01	6.74	0.18	6.66	0.22
MCC950	8.5	0.004	7	0.1	7.22	0.06
*NBC inflammasome inhibitor series*						
NBC6	5.2	6.3	5.96	1.17	5.33	4.67
NBC11	5.8	1.66	5.84	1.44	5.53	2.93
NBC12	6.3	0.48	6	0.99	5.53	2.98
NBC13	6.4	0.41	6.06	0.87	5.69	2.02
NBC19	7.1	0.08	5.93	1.18	6.07	0.85
*Caspase-1 inhibitor*						
VX-765	7.8	0.01	7.15	0.07	7.3	0.05

THP1 cells were primed with 1 µg/ml LPS overnight, treated with inhibitors for 30 minutes and further stimulated with nigericin (10 µM, 1 h). ASC specks were analysed (Table 2) and supernatants used for MSD quantification of IL-1β release. pIC50 and IC50 values were determined by normalisation to vehicle-treated controls, and obtained from at least 3 independent experiments. Assays were performed using microglial cultures primed with LPS (1 μg/ml, 3 h), treated 15 minutes with the inhibitors, and further activated with ATP (5 mM, 1 h) or nigericin (10 μM, 1 h). pIC_50_ and IC_50_ values were determined by vehicle-normalised ELISA quantification of IL-1β, and obtained from at least 3 independent cultures (litters).

**Figure 3:**
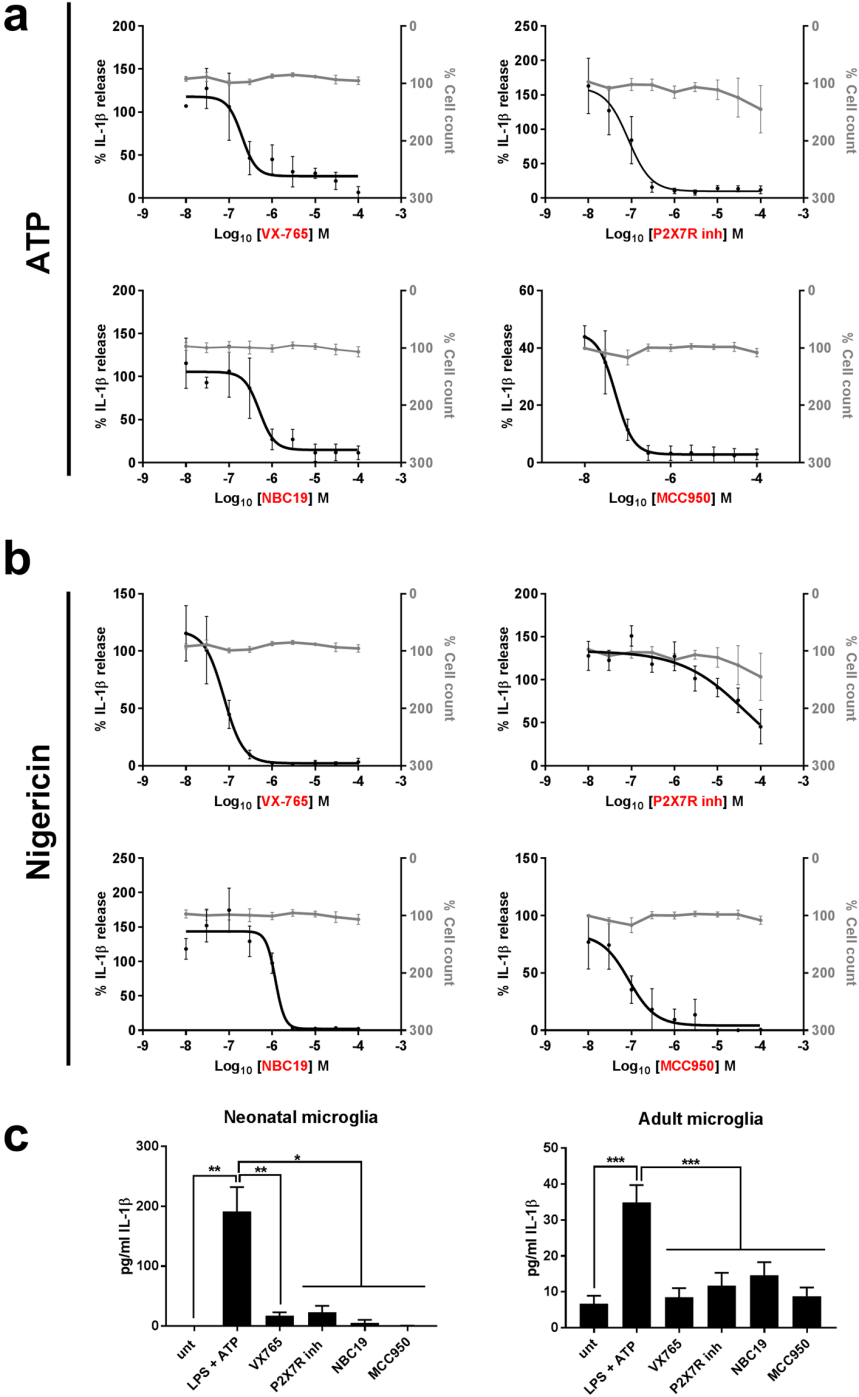
Inhibition of IL-1β secretion in neonatal and adult microglia. 50 × 10^4^ cells were treated with LPS (1 μg/ml, 3 h), then with the selected inhibitors (VX-765, P2X7R inhibitor (compound 26 from reference^19^), NBC19 and MCC950, 10 μM for 15 min), and then activated with ATP (**a**, 5 mM, 1 h) or nigericin (**b**, 10 μM, 1 h). Graphs are presented showing cell death curves (in grey) and the inhibition of IL-1β release (in black) from neonatal cultures of microglia (a and b). The four inhibitors tested significantly reduced the secretion of IL-1β both in neonatal microglia and adult microglia (**c**). Statistical significance (vs. LPS + ATP): *p < 0.05; **p < 0.01; nd: not-detectable. Scale bar = 200 µm.

We next tested the molecules in cells relevant to CNS disease. Cultured mouse neonatal microglia were prepared as described previously^27^ where inflammasome responses are known to be microglia dependent^27,28^. Cells were primed with LPS (1 μg/ml, 3 h), then incubated with the inhibitors using a 10-point concentration response curve, before incubation with either nigericin (10 μM, 1 h), or ATP (5 mM, 1 h), in a minimum of four separate experiments. IL-1β release was measured as the endpoint. The best inhibitor from the diarylsulfonylurea series was again MCC950 with an IC_50_ of 60 nM against ATP-induced IL-1β release (Table 3). The P2X7 antagonists had no effect against nigericin-induced IL-1β release but were effective against ATP with AZD9056 and compound 26 (from^19^) both inhibiting ATP-induced IL-1β release with an IC_50_ of 30 nM (Table 3). NBC19 was again the most effective of the NBC series with an IC_50_ of 850 nM against ATP-induced IL-1β release (Table 3). VX-765, the caspase-1 inhibitor, inhibited ATP-induced IL-1β release with an IC_50_ of 50 nM (Table 3). There is debate in the literature as to whether responses are conserved between cultured neonatal microglia and adult microglia^29,30^. Thus we isolated primary adult mouse microglia as previously described^31^, and treated them as above (*e.g*. LPS, 1 μg/ml, 3 h followed by 15 min of inhibitor, followed by 5 mM ATP for 1 h). At this stage we selected the 4 best compounds across the respective classes (*i.e*. the P2X7 inhibitor compound 26 from^19^, MCC950, NBC19, and VX-765; dose responses to ATP and nigericin shown in Fig. 3a and b respectively). ATP-induced NLRP3 inflammasome activation and IL-1β secretion was conserved in isolated adult microglia and the 4 inhibitors (all tested at 10 μM, n = 10) effectively inhibited IL-1β release (Fig. 3c).

## Discussion

Given the established role for IL-1 in human disease^32^, and preclinical evidence showing the involvement of NLRP3 in many major diseases including Alzheimer’s disease^11,12,33^, diabetes^34^, and cardiovascular disease^14^, the NLRP3 inflammasome is emerging as a potential drug target. Here we have characterised and compared a number of compounds known to be inhibitory across the various points in the NLRP3 pathway (Fig. 1). By testing the compounds side by side in validated assays we have been able to show that there are potent inhibitors for a number of steps of the NLRP3 pathway. The P2X7 inhibitor compound 26 is of interest for use in CNS models of disease as it is known to have high permeability across the blood brain barrier^19^, and we show here that it is very effective at inhibiting inflammasome activation and IL-1β release against ATP (Tables 2 and 3). Compound 26 did not inhibit nigericin induced NLRP3-dependent IL-1β release (Tables 2 and 3) confirming it as a potent and selective inhibitor of the P2X7-NLRP3 axis. As reported previously, MCC950 is a potent and selective inhibitor of NLRP3 inflammasomes *in vitro* and *in vivo*^22^ (Tables 2 and 3). Caspase-1 activation is an outcome of inflammasome activation and so we predicted the caspase-1 inhibitor VX-765 would be effective at inhibiting IL-1β release but not ASC speck formation which was confirmed (Tables 2 and 3). That VX-765 did not inhibit ASC speck formation confirmed that it was targeting caspase-1 specifically downstream of inflammasome activation (Table 2, Fig. 2). The comparison between the diarylsulfonylurea series^20–22^, and our NBC series^24^, showed that the best diarylsulfonylureas were in general more potent, and the most potent of them, MCC950, was the most active compound tested (Tables 2 and 3). Our recent work described NBC6 (with an IC_50_ of 570 nM) as an effective inhibitor of the NLRP3 inflammasome *in vitro* and *in vivo*^24^. We report here that NBC19 has improved activity, with a significantly improved IC_50_ of 60 nM in the ASC inflammasome speck assay in differentiated THP1 cells (Table 2). Thus we have characterised a ‘tool kit’ for dissecting inflammasome dependent responses identifying potent and well characterised reagents.

Here we established 2 protocols for assaying the effects of the inhibitors. The assay developed using the THP1-Cerulean cells^9^, was particularly sensitive, consistently yielding lower IC_50_ values than the primary glial cultures (Tables 2 and 3). Given that the IC_50_ established from the differentiated THP1 cells for IL-1β release more or less mirrored the IC_50_ obtained in the ASC speck assay, we think that the difference between the THP1 cells and the glia likely reflects the inherent variability between primary cultures when compared to using a pure clonal cell population. We also established that inflammasome responses are robust and comparable in acutely isolated primary microglia from the adult brain (Fig. 3c), giving confidence to the data generated using neonatal microglia.

It is important to note, that while our research has focussed on targeting the NLRP3 inflammasome in innate immune cells, NLRP3 is also important in non-immune cell function. For example, the NLRP3 inflammasome is present in epithelial cells in many tissues and is known to be involved in many physiological processes^35^. There is evidence that epithelial cell NLRP3 also regulates caspase-1-dependent release of pro-inflammatory cytokines such as IL-1β (e.g.^36^), but it is also reported to have other functions such as regulating the activation of sterol regulatory element binding proteins (SREBPs) which repair the plasma membrane of epithelial cells in response to pore forming bacterial toxins^37^. Thus the ‘toolkit’ described here will also be of value to scientists studying NLRP3 function in non-immune cells such as epithelial cells.

In summary, the data provided here give comparable quantitative data on inhibitors of the NLRP3 inflammasome pathway and identify their selectivity across the particular points of intervention, identifying them as a valuable tool kit for interrogating inflammasome dependent responses in cell based models.

## Materials and Methods

### Inhibitors

The NBC molecules were synthesised and prepared at the University of Manchester as described previously^24^. MCC950 (Sigma-Aldrich, Dorset, UK) and VX-765 (Selleckchem, Munich, Germany) were purchased. All other compounds in the P2X7 and diarylsulfonylurea series were synthesised following the relevant published literature either in house at LifeArc or at GVK Biosciences, Bengaluru, India.

### THP1 culture and differentiation

ASC-Cerulean expressing THP1 cells were grown in RPMI, 10% FBS and 1% Pen/Strep. Cells were differentiated using 0.5 µM PMA for 3 h and used for assays the next day.

### NLRP3 inflammasome activation in THP1 cells

ASC-Cerulean expressing THP1 cells were pre-incubated with compound (1% final DMSO) for 30 min before being stimulated with 10 µM nigericin for 1 h. Pre-differentiated cells were treated with the pan caspase inhibitor Z-VAD-FMK (Promega, Southampton, UK; 50 µM, 30 min) during compound incubation. Supernatants were collected for quantification of IL-1β using mesoscale Tissue Culture Kit (K151AGB) and cells were fixed for the speck assay.

### Speck assay and analysis

ASC-Cerulean expressing THP1 cells were fixed with 4% formaldehyde for 15 min at room temperature, washed with PBS and stained with HCS Red (Invitrogen, Loughborough, UK) to visualize nuclei. Images were acquired on the GE IN Cell 2000 and analysed in Workstation to count both nuclei and cerulean-labelled ASC specks.

### Preparation of neonatal glial cultures

Mice (C57BL/6 strain) were maintained under standard laboratory conditions (20 ± 2 °C, 12-h light cycle, humidity of 40–50%, 12 h light cycle, *ad libitum* access to water and standard rodent chow). All procedures were performed by appropriate personal and under project licences, in accordance with the Home Office (Animals) Scientific Procedures Act (1986) and approved by the Home Office and the local Animal Ethical Review Group, University of Manchester. Each litter was considered as an n number, and a minimum of 3 litters was used to test each compound.

Brains were harvested from 3–4 days old mice under aseptic conditions, and olfactory bulbs, cerebellum, and meningeal layers, were gently removed. Brain tissue was mechanically digested, centrifuged, and cells resuspended in media and seeded on cell culture flasks. Cells were maintained in Dulbecco’s Modified Eagle’s Medium (DMEM, Sigma-Aldrich), 10% fetal bovine serum (FBS, Life Technologies, Warrington, UK), 100 U/ml penicillin and 100 μg/ml streptomycin (Sigma-Aldrich). Media was changed every 4–5 days until cells reached 80% confluence (around day 10–13 *in vitro* (div)). Cells were then trypsinized (0.5% Trypsin EDTA, 2 min at 37 °C, Sigma-Aldrich) and gently scraped, counted and seeded on plates (1.7 × 10^5^ cells/ml). After 2–3 days, cells were ready for further experiments.

### Preparation of adult microglia

Adult C57BL/6 mice were perfused with ice-cold Hank’s Balanced Salts Solution (HBSS) and brains kept in cold HBSS. After removal of cerebellum and meningeal layers, brains were diced, and enzymatically processed using as per manufacturer’s instructions using a MACS Neural Tissue Dissociation Kit (P) (Miltenyi Biotech, Bisley, UK). Brains were then mechanically homogenised using a Dounce homogeniser, and myelin removed from the resulting cell suspension using a one-step 30% Percoll gradient^31^. Microglia were then isolated using magnetic CD11b + beads (Milteny Biotech), and seeded on poly-L-lysine coated plates (~1.7 × 10^5^ cells/ml), and maintained with DMEM (with 10% fetal bovine serum and 1% penicillin streptomycin) supplemented with 10 ng/ml of recombinant mouse M-CSF (R&D systems, Abingdon UK), and 50 ng/ml of recombinant human TGF-β. Media was changed at 3 days, and cells treated after 7 days in culture.

### Inflammasome activation assays in glia

Fresh media (DMEM, 10% FBS, 100 U/ml penicillin and 100 μg/ml streptomycin) was added before starting the treatments. Cells were treated with gel filtration chromatography purified LPS (1 µg/ml, 3 h, *E.coli* O26:B6, L2654, Sigma-Aldrich) and then the media was changed to serum free media before the addition of drugs that were dissolved in DMSO (except MCC950, in PBS), and were added as a 10 point dose response from 0.01–100 µM (compounds shown in Table 1). After 15 min incubation with the drugs (or vehicle), the NLRP3 inflammasome was activated as follows by adding ATP (5 mM) or nigericin (10 µM) for 1 h. Supernatants were collected and used for further analysis of cell death and IL-1β release.

### Lactate dehydrogenase measurement of cell death

Lactate dehydrogenase (LDH) release was used as a measure of cell death using a CytoTox 96® Non-Radioactive Cytotoxicity Assay (Promega). Following manufacturer’s instructions, absorbance at 490 nm was measured and converted into cell death values.

### IL-1β measurements

#### THP1 cells

Secretion of IL-1β was quantified using mesoscale Tissue Culture Kit (K151AGB) following the manufacturer’s instruction.

#### Microglial cells

Secretion of IL-1β was quantified by ELISA using DuoSet® kits (R&D Systems) following the manufacturer’s instructions.

### Statistical analysis

#### THP1 cells

For both readouts, speck assay and IL-1β quantification, half maximal inhibitory concentration (IC_50_) of drugs was determined by fitting the data to a four parameter logistic equation (Nonlinear Regression, Sigmoidal, 4PL) in GraphPad Prism version 7.03. Mean and SEM values are plotted from at least 3 experiments.

#### Glia

For each assay IL-1β levels were calculated against a four-parameter logistic (4-PL) curve fit, using GraphPad Prism version 7.00 for Windows, GraphPad Software (USA). All values are expressed as a percentage of vehicle conditions or as mean ± standard error of the mean (SEM), and a minimum of 3 litters (n = 3) was used for each tested compound. The half-maximal inhibitory concentration (IC_50,_ μM) for each drug was determined by fitting the data to a four-parameter logistic equation using GraphPad Prism. One way ANOVA was performed on IL-1β values for the adult and neonatal microglia experiments (comparing vs. LPS + stimulus). Holm-Sidak *post hoc* tests were performed if statistical significance (p value < 0.05) was achieved, and indicated in the graphs as follows: *p < 0.05; **p < 0.01; ***p < 0.001.

### Data availability

The data that support the findings of this study are available from the corresponding author on request.
